# Interactions between sleep, inflammation, immunity and infections: A narrative review

**DOI:** 10.1002/iid3.70046

**Published:** 2024-10-17

**Authors:** Thijs Feuth

**Affiliations:** ^1^ Department of pulmonary diseases and Allergology Turku University Hospital Turku Finland; ^2^ Pulmonary Diseases and Allergology, Faculty of Medicine University of Turku Turku Finland

**Keywords:** cytokines, immunity, infections, inflammation, interleukin‐1, long‐COVID, parasitic infections, sleep, sleep apnea, TNF, viral infections

## Abstract

**Background:**

Over the past decades, it has become increasingly evident that sleep disturbance contributes to inflammation‐mediated disease, including depression, mainly through activation of the innate immune system and to an increased risk of infections.

**Methods:**

A comprehensive literature search was performed in PubMed to identify relevant research findings in the field of immunity, inflammation and infections, with a focus on translational research findings from the past 5 years.

**Results:**

Physiological sleep is characterized by a dynamic interplay between the immune system and sleep architecture, marked by increased innate immunity and T helper 1 (Th1) ‐mediated inflammation in the early phase, transitioning to a T helper 2 (Th2) response dominating in late sleep. Chronic sleep disturbances are associated with enhanced inflammation and an elevated risk of infections, while other inflammatory diseases may also be affected. Conversely, inflammation in response to infection can also disrupt sleep patterns and architecture. This narrative review summarizes current data on the complex relationships between sleep, immunity, inflammation and infections, while highlighting translational aspects. The bidirectional nature of these interactions are addressed within specific conditions such as sleep apnea, HIV, and other infections. Furthermore, technical developments with the potential to accelerate our understanding of these interactions are identified, including advances in wearable devices, artificial intelligence, and omics technology. By integrating these tools, novel biomarkers and therapeutic targets for sleep‐related immune dysregulation may be identified.

**Conclusion:**

The review underscores the importance of understanding and addressing immune imbalance related to sleep disturbances to improve disease outcomes.

## 1 INTRODUCTION

Links between sleep and infections have been proposed since ancient times, but the first scientific evidence of the interaction between sleep and immunity dates from the late 1970s, when Jan Palmblad et al demonstrated that sleep loss led to altered lymphocyte DNA synthesis after stimulation in vitro.^1^ Since then, numerous studies have demonstrated a reciprocal relationship between sleep and immunity. Over the past decades, it has become increasingly evident that sleep disturbance contributes to inflammation‐mediated disease, including depression, mainly through activation of the innate immune system and to an increased risk of infections.^2^ On the other hand, the inflammatory response to infections may interfere with physiological sleep.^3^ This review summarizes the current understanding of the complex relationships between sleep, immunity, inflammation and infections.

### 1.1 Communication between the central nerve system and the peripheral immune system

The immune compartment within the brain consists of microglia and infiltrating immune cells.^4^ Other cells in the brain, such as astrocytes, oligodendrocytes and nonparenchymal macrophages, also express receptors related to innate immunity and may be capable of antigen presentation and of the production of cytokines.^5^ Recent studies have identified interactions between the central nerve system (CNS) and the peripheral immune system, involving meningeal lymphatics, cytokines, chemokines, and neurotransmitters, alarmins released by neurons, and highly permeable and fenestrated capillaries in circumventricular organs (CVOs).^5^ Furthermore, the neurotransmitter acetylcholine, which is essential in communication between neurons in the central and peripheral nerve system, also plays a role in regulation of immunity via the cholinergic anti‐inflammatory pathway, which communicates between the nervous and immune systems as part of a well‐characterized neural circuit termed the inflammatory reflex, and which is involved in several diseases.^6^

### 1.2 Innate immunity

The innate immunity, which serves as the first line of defence against infections, includes various cell types such as dendritic cells, macrophages, mast cells and natural killer (NK) cells. These immune cells are activated through pattern recognition receptors (PRR) on the cell surface, which detect pathogen associated molecular patterns (PAMPs) or damage‐associated molecular patterns (DAMPs). PRRs include Toll‐like receptors (TLRs) and Nod like receptors (NLRs). Activation of innate immune cells may also occur through detection of cells missing certain molecules that are expressed by healthy cells.^7^ This triggers a signalling cascade, which is characterized by the activation of intracellular transcription factors such as nuclear factor‐κB (NF‐κB) and activator protein 1 (AP‐1), leading to the production of Pro‐inflammatory cytokines like interleukin (IL)‐1β, tumour necrosis factor (TNF) and interferon (IFN)‐α.^2^, ^7^ The type of cytokine response depends on many factors, including the route of activation.^7^

The complement cascade, which forms a key component of the innate immune system, can be activated through three primary pathways: the lectin pathway, which is driven by recognition of PAMPs by mannose binding lectin (MBL), the classical pathway, which is initiated when C1q binds to an antibody–antigen complex, and the alternative pathway, upon spontaneous hydrolysis of C3. Once activated, the complement system promotes inflammation, lysis of pathogens and opsonization, by cleaving complement proteins into active components such as C3a, C4a, and C5a. These components rapidly activate several immune functions including opsonization, chemotaxis, adhesion, phagocytosis, immune modulation and immune complex clearance.^8^, ^9^ Recently, high‐throughput multi‐omics techniques have contributed to an understanding that complement exert functions beyond its traditional realm in innate immunity, including regenerative and immunometabolic functions via a crosstalk with multiple pathways both in the intracellular as in the extracellular space.^9^

Sleep interacts with the complement system. At night, plasma levels of complement factors C3 and C4 decrease, while complement activation is reflected by increased plasma C3a levels and while the regulatory activity of C5a decreases.^10^ Sleep deprivation is associated with increased levels of complement factors C3 and C4 as well as increased levels of circulating antibodies.^11^

### 1.3 Adaptive immunity

Interactions between sleep and immunity is not restricted to the innate immune system. For instance, the circadian variation of TLR‐9 responsiveness at the time of immunization may affect the magnitude of adaptive immune responses.^12^ Neutrophils play a key role in innate as well as in adaptive immune responses. Sympathetic nerve system‐mediated circadian changes in CXCL12 promotes circadian release of neutrophils from matured neutrophils into the bloodstream.^13^ The function of neutrophils may be impaired in short‐term sleep deprivation, resulting in decreased levels of peripheral CD4 + T cells through chances in the T helper 1 (Th1) and T helper 2 (Th2) chemokines balance.^14^ Furthermore, the migration of lymphocytes shows a circadian variation, with homing to lymph nodes peaking at the onset of night.^15^ Migration of T cells towards the lymph nodes during sleep is at least partly mediated by CCL19.^16^ Intrinsic mechanisms, possibly involving the NF‐κβ pathway, play a role in the regulation of the circadian rhythm of T cell responses.^17^ The circadian rhythm in T cell migration and function may have clinically relevant sleep‐mediated effects. In a study on 27 healthy individuals, sleep after combined vaccination for hepatitis A and hepatitis B was associated with a markedly increased response to vaccination,^18^ and similar findings were reported by others.^18^, ^19^ However, in a study among 360 healthcare workers, antibody levels after vaccination against COVID‐19 did not correlate with sleep quality after adjusting for confounders, and no correlation with breakthrough infection was found.^20^ Similarly, objectively measured peri‐vaccination sleep failed to predict COVID‐19 breakthrough infection in a large cohort of 7,848 fully vaccinated individuals with sufficient sleep data.^21^

### 1.4 Pro‐inflammatory Th1 activation in early sleep

In physiological sleep, a downregulation of the hypothalamus‐pituitary‐adrenal (HPA) axis to the 24‐h minimum and downregulation of the sympathetic nervous system (SNS) is associated with drops in levels of cortisol, epinephrine and norepinephrine, while the levels of growth hormone (GH), prolactin, melatonin and leptin peak. The synergetic effects of these changes are Pro‐inflammatory, promoting a Th1 inflammatory response with the production of IL‐1β and TNF in the early sleep.^22^

Pro‐inflammatory markers IL‐1β and TNF have pro‐somnogenic properties, enhancing slow‐wave sleep (SWS) in early night.^23^, ^24^ In the brain, TNF is expressed by microglia, astrocytes and neurons, and is involved in regulation of essential functions in physiological as well as in pathological conditions, including sleep regulation.^25^ In human studies, the TNF is typically measured from the serum, together with associated cytokines such as IL‐1β. Systemic TNF seems able to promote brain TNF production, but may also be transported from blood to brain.^25^

Pro‐somnogenic effects of cytokines primarily influence non‐rapid eye movement (NREM) sleep regulation, while the effect on rapid eye movement (REM) sleep may be reversed, depending on the levels.^26^ Current evidence indicates that acute mild inflammation may suppress REM sleep, while stronger inflammation supresses both NREM and REM sleep.^3^ The inflammation‐associated increase of NREM sleep may favour recovery from infectious disease.^27^ Inflammation related to malignant disease, neurodegenerative diseases, metabolic diseases and auto‐immune disease show similar bidirectional interactions with sleep, while immunity may be involved in the inverse comorbidity phenomenon that can be observed between certain cancers and neurodegenerative diseases.^3^, ^28^

The use of anti‐TNF monoclonal antibody infliximab is associated with improved sleep disturbances in rheumatoid arthritis (RA) independent of joint discomfort amelioration.^29^ Furthermore, a randomized study revealed that TNF blockade improved sleep continuity in patients with treatment‐resistant depression and enhanced inflammation.^30^ Stimulation of the vagus nerve may also inhibit production of TNF, IL‐1β and IL‐6 and attenuate disease severity in rheumatoid arthritis via the inflammatory reflex.^31^

### 1.5 Immune dynamics with Th2 predominance in late sleep

Late sleep, when REM sleep dominates, is associated with rapid increase in HPA axis activity after the minimum in early night, resulting in increasing levels of cortisol and a switch to Th2 cytokine production. Cytokines IL‐4, IL‐10, and IL‐13 exert anti‐somnogenic functions in addition to their regulatory roles in immunity. IL‐4 inhibits activation of macrophages, induces a B class switch to IgA and decreases the production of Th1 cell while promoting Th2 inflammation. While this IL‐4‐mediated inflammation may be a beneficial in helminth infection, it also plays a pivotal role in asthma, along with IL‐10 and IL‐13. Indeed, asthma symptoms predominate at night and early morning, while presently available data suggest that there is a causal relationship between poor sleep and Th2‐mediated asthma.^32^ Furthermore, IL4‐ and IL‐13 antibody blockers improve sleep in patients with airway or skin disease, although it is unclear whether the effect is independent of the improvement of the underlying disorder or the reduction of corticosteroid treatment, which may interfere with sleep.^33^, ^34^, ^35^, ^36^

### 1.6 The associations of histamine and melatonine with sleep and immunity

Histamine and melatonin play have essential roles in the regulation of sleep and wakefulness, mainly through NF‐kB‐mediated interaction with core clock genes, as well as in immune regulation.^37^ Histamine, which is associated with wakefulness, is a potent mediator of inflammation through pleiotropic effects via different receptors that are widely expressed throughout the body on many different cell types. Binding to histamine 4 receptors on eosinophils increased the expression of macrophage‐1 antigen (Mac1) and intracellular adhesion molecule 1 (ICAM‐1), resulting in inflammation. By interaction with H1R, histamine induces the production of inflammatory cytokines such as IL‐6 by macrophages and IFN‐γ through activation of Th1 cells.^38^ Similarly, melatonin, which peaks around 3 to 4 AM, has modulating effects on innate immunity and on T cell‐mediated immunity, while several studies indicate that melatonin may exert antiviral effects.^39^, ^40^, ^41^ In turn, the altered immune state in infections and malignant disease disrupts the circadian dynamics of melatonin.^41^

### 1.7 Sleep deprivation

Poor sleep is associated with excessive TNF plasma levels in a number of conditions including sleep apnea, insomnia, excessive daytime sleepiness, and in many inflammatory conditions such as infections, rheumatological disease, and malignancies. Prolonged sleep disturbance leads to activation of the HPA axis and the SNS axis.^3^ In animal models, REM sleep deprivation induced expression of IL‐1β genes in the hypothalamus and increased neuro‐inflammation.^29^, ^42^ In humans, chronic sleep deprivation is associated with increased levels of IL‐1β and TNF as well as with downstream inflammation markers such as IL‐6 and C‐reactive protein (CRP).^3^, ^43^ Moreover, successful treatment of insomnia results in downregulation of inflammation.^3^

A wide range of studies, including cross‐sectional, population‐based, as well as experimental viral challenges studies have demonstrated that sleep deprivation is associated with an increased risk of infections of the respiratory tract and beyond, and immune responses after vaccination may be reduced.^27^ This is most likely related to the enhanced inflammatory profile observed in these individuals.

### 1.8 Nocturnal hypoxemia

In sleep apnea, not only sleep deprivation may interfere with immunity, but also intermittent hypoxemia and tissue hypoxia. Indeed, hypoxemia leads to adaptation through a number of signalling pathways including hypoxemia inducible factor (HIF), Nuclear factor‐κB (NF‐κB) and cyclic AMP response element binding protein (CREB), which also may lead to dysfunctional immune responses.^44^ However, the response to hypoxemia differs depending on the cell type.^45^ Furthermore, it has been demonstrated that hypoxemia suppresses type I IFN signalling in acute lung injury, thereby enhancing neutrophil‐mediated inflammation in the lung through reduced accumulation of monocyte‐derived macrophages.^46^ Mice display significant increases in IL‐6, TNF and IL‐1β after exposure to hypoxemia.^47^ Thus, intermittent hypoxemia can lead to innate immune activation.

The effects of hypoxemia on the adaptive immunity are different. HIF‐1 impairs T cell functions through the production of adenosine. Hypoxemia may impair T‐cell proliferation and downregulate T‐cell activation by upregulation od checkpoint mechanisms through the hypoxia‐adenosine pathway and subsequent activation of the PD‐1‐ and CTLA‐4‐dependent immunosuppression.^48^

### 1.9 Inflammation and infection risk in sleep apnea

Sleep apnea patients have increased levels of pro‐inflammatory cytokines such as TNF, IL‐6, IL‐8 and IL‐1β and an increased activation of the complement system.^49^, ^50^, ^51^ Furthermore, it has been demonstrated that systemic immune‐inflammation is correlated to the severity of hypoxemia in sleep apnea.^51^, ^52^ Good compliance to treatment with CPAP, which effectively treats hypoxemia, is associated with a downregulation of the inflammatory state in sleep apnea patients.^53^ Together, the available evidence points at a pro‐inflammatory state of the innate immunity and decreased T‐cell‐mediated specific immunity in sleep apnea patients, while this immune dysfunction is at least partially reversible by adequate treatment.^45^ Indeed, patients with sleep apnea have higher rates of pulmonary infections and with more severe disease, while current evidence suggest that treatment of sleep apnea may normalize the infection risk.^54^, ^55^ Similarly, untreated sleep apnea is associated with exacerbation risk in chronic airway disease such as asthma and COPD, with likely roles for enhanced systematic inflammation and airway tissue inflammation through airway collapse, hypoxemia, oxidative stress, and gastroesophageal reflux.^54^, ^56^

Furthermore, sleep apnea is associated with common life‐style associated comorbidities such as obesity and diabetes mellitus, which are also associated with immune activation, with possible roles for adipocytes and gut microbiota.^57^, ^58^

### 1.10 Common viral infections

Acute rhinovirus and influenza virus infections are associated with self‐limiting sleep disturbances such as increased proportion of sleep spent in NREM, while REM is reduced and decreased consolidated sleep time and sleep efficiency. In these conditions, the acute increase of pro‐inflammatory cytokines IL‐1β and TNF, and their pro‐somnogenic properties, may fully explain the altered sleep pattern.^59^

### 1.11 Epstein barr‐virus

Tiredness and excessive sleeping are frequent symptoms associated with mononucleosis Epstein‐Barr virus (EBV) primary infection in puberty and young adults, yet the immune mechanisms involved remain poorly understood.^60^ Acute infection mounts innate immune responses mainly through activation of signalling pathways mediated by TLR2 on host immune cells, which results in the secretion of TNF, IL‐1β, IL‐6 and IL‐10.^61^ EBV deploys several strategies to evade immunity, including inhibition of apoptosis, cell proliferation, and the inhibition of proteasomes, which allows it to establish life‐long persistence in most individuals, almost exclusively in B lymphocytes.^62^ In its latency, EBV successfully inhibits inflammation and immunity through interaction with different cell lines, including cytotoxic T cells, B cells, and NK cells.^63^ Even though tiredness and excessive sleeping are thought to be the result of prolonged immune disturbances, the etiologic mechanism is poorly studied. Polysomnography data from patients with infectious mononucleosis is scarce and non‐conclusive. A study performed in 1986 found no changes in sleep architecture in 12 patients with chronic daytime sleepiness related to mononucleosis.^64^ Nevertheless, although the condition can last for months, sleepiness is generally a self‐limiting symptom in EBV and there is no evidence for interventions.^63^

### 1.12 HIV

Sleep disturbances are also frequently reported in patients living with HIV (PLWH), including increased sleep latency and sleep fragmentation.^65^ In early stages of HIV infection, patients may suffer from insomnia, while polysomnography has revealed a relative shift to N3 sleep in the latter part of night.^66^ Sleep disturbances increase with the severity of HIV infection.^65^ However, sleeping disorders may also be related to medications, such as zidovudine, which may cause insomnia, and nevirapine and efavirenz which may cause sleep disruption and vivid dreaming.^66^ Sleeping disorders related to dolutegravir are also reported, but are less common. Morning dosing of dolutegravir may be sufficient to solve sleeping disorders in these patients.^67^

### 1.13 Long‐COVID

A proportion of patients report prolonged sleeping disturbances after acute COVID‐19. In a study from Brazil on 189 long‐COVID patients, 22% reported insomnia and 3% reported excessive sleepiness, and these conditions were associated with corticosteroid use.^68^ A case‐control study on 17 long‐COVID patients with insomnia had a pattern of chronic insomnia, which was similar to patients with chronic insomnia without long‐COVID.^69^ A study on 122 recovered COVID‐19 patients found no meaningful difference in total sleep duration in comparison to controls, but a small decreased deep sleep times (123 *vs.* 128 min) as measured by a wearable device.^70^

One study revealed sleep apnea by polysomnography in 60% of 20 patients with long‐COVID.^71^ Another study found sleep efficiency <80% in 50% of patients with long‐COVID, while a pattern consistent with obstructive sleep apnea was observed in 35% of 42 patients undergoing polysomnography.^72^

These studies underline the importance of polysomnography in prolonged symptoms after acute COVID‐19. However, other studies have revealed that patients with prolonged symptoms may display increased levels of the inflammatory markers IL‐1β, IL‐6 and TNF, suggesting that an immune imbalance may also be involved in prolonged symptoms after COVID‐19.^73^

### 1.14 Other viral infections

A variety of other viral pathogens may cause changes in sleep, such as the induction of slow wave sleep (SWS). These include hepatitis B, hepatitis C and cytomegalovirus. It is believed that the inflammatory response mediates the effects on sleep through induction of IL‐1β, IL‐6, TNF and type I interferon. However, related sleep patterns and potential etiologic mechanisms are poorly addressed in medical research.^2^

### 1.15 Encephalitic lethargica

In 1917–1925, an epidemic of an encephalitis‐like disease causing delirious somnolence occurred in Europe. The cause of encephalitis lethargica, also referred to as Von Economon tauti, is thought to be related to immunity to a pathogen of viral origin, but the definite etiology remains still to be elucidated.^74^

### 1.16 Lyme borreliosis

Although commonly reported, sleep disturbances among patients with Lyme disease are poorly understood. In 1995, a study performed on 11 patients with serologic confirmation of late Lyme disease revealed sleep related complaints among all, with excessive daytime somnolence in 73%, with a decreased length of uninterrupted occurrences of stage 12 and stage 4 NREM sleep.^75^ Patients with sleep disturbances in early Lyme disease generally recover within 6 months from antibiotic treatment, while 10–15% develop posttreatment Lyme disease syndrome, and still report sleeping disturbances after 12 months.^76^

### 1.17 Sleeping sickness

Disturbed sleep is one of many symptoms of Human African trypanosomiasis (HAT), more commonly known as sleeping sickness. The disease results from infection with the protozoan parasite *Trypanosoma brucei*, which is transmitted by the tsetse fly in foci in some rural, impoverished areas of Sub‐Saharan Africa.^77^ The early, haemolytic phase is defined by the restriction of infection to the blood and lymph systems, which may cause chronic or intermittent fever, headache, and skin lesions and lymphadenopathy. The second, meningo‐encephalitic stage is characterized by invasion of the CNS by entering the brain via CVOs and the choroid plexus in a T‐cell dependent matter.^78^, ^79^ In this stage, sleep disturbances include alterations of the sleep architecture, characterized by sleep‐onset rapid eye movements (SOREM), and in a later phase reversed sleep, ie daytime somnolence and nocturnal insomnia, as well as narcoleptic crises, and uncontrollable urges to sleep.^77^, ^80^, ^81^ Several pathogenetic mechanisms may contribute to the sleep‐wake disruption, including glial cell activation, interference with the biological clock, and alterations in the production of cytokines and prostaglandins.^81^ If left untreated, the condition is fatal. Oral treatment options with either fexinidazole or acoziborole have recently been established.^82^ After treatment, sleep alterations may disappear, but improvements may take several months.^81^

### 1.18 Other parasitic disease

A number of parasite infections are characterized by circadian rhythms, with typically high blood parasite levels in *Wuchereria bancrofti* (in lymphatic filariasis), plasmodium merozoites (in malaria) and leishmania amastigotes at night and periodic episodes of fever in malaria, occurring every 24, 48, or 72 h. Thereby, parasites may benefit from the Th1 predominance during early night, while the eosinophilic IL‐4 response dominates in the late phase of sleep.^83^ However, the relationships between human and parasite circadian rhythms remains poorly understood and may also involve the route of transmission and the activity of its vectors, such as nocturnal biting activity of the sand fly in leishmaniasis, of the Anopheles mosquito in malaria and Anopheles as well as Culex mosquitos in lymphatic filariasis.^83^ However, *W. bancrofti* is transmitted by Aedes mosquitos in the Pacific and there, circulating levels of *W. bancrofti* is typically higher in the afternoon, resembling the peak feeding behaviour of its vector.^83^, ^84^ Thus, the relation between circadian rhythms of parasitic disease is probably multifaceted, and may include adaptation to the circadian immune dynamics in the host.

### 1.19 The human microbiome

Recent studies have also highlighted a prominent role of the microbiota in the gastrointestinal tract in the regulation of immunity. These microbiota and their metabolites affect the risk and course of infections through several mechanisms, including regulation of innate and adaptive immunity.^85^ The gut microbiota also plays a role in the coordination of the differential innate immunity activity during day and night, which synchronizes with feeding rhythms and thereby with exogenous microbial exposure.^86^ The respiratory tract is also inhabited by communities of microbes, while their role in respiratory immunity and infections is currently being studied.^87^

Sleep disorders are associated with dysbiosis of the microbiome of the gut and the respiratory tract, while the available evidence suggests a bidirectional relationship between the composition of microbiota and the sleep disorders.^88^ In sleep apnea, intermittent hypoxia, hypercapnia, complement activation and sleep fragmentation may contribute to dysbiosis, while data from several studies suggest that alteration of the microbiome with probiotics, prebiotics and microbiota transplantation could improve sleep.^89^

### 1.20 Other factors associated with sleep and infections

Sleep may also play a role in infection risk beyond the immune system, for instance through nocturnal aspiration. Horizontal position in sleep predispose to aspiration while the cough reflex is suppressed and may thereby lead to pneumonia.^90^ Drugs with sedative effects, such as opioids increase the risk of aspiration pneumonia.^91^ Bacterial translocation in micro‐aspiration from the oesophageal to the respiratory tract may cause changes in the lung microbiome persistent subclinical infection, which may act as a cofactor in infection‐associated conditions such as acute exacerbation of chronic obstructive lung disease, bronchiectasis and cystic fibrosis, but also in other respiratory diseases such as idiopathic pulmonary fibrosis.^92^

Several other factors may be associated with sleep and infections. For instance, high age is associated with reduced sleep and with the development of sleeping disorders, while infection risks in general increase dramatically in elderly patients.^3^ Similarly, obesity and related conditions are associated with increased inflammation, aspiration, and pulmonary infections, while obesity is also associated with sleep apnea and insomnia, while physical activity may be protective.^93^ Comorbidities, the use of substances including alcohol and sleep disparities such as poverty, poor education, ethnicity and poor diet, are all known to affect the risk of infections.^94^, ^95^

### 1.21 Prospects on research and clinical implications

The importance of sleep in health and homeostasis has been recognized since ancient times, but the underlying biologic and immune mechanisms have only been discovered over the past decennia. New technological advances have the potential to further accelerate our understanding of the interactions between sleep, inflammation, immunity and infections. Wearable devices measuring sleep patterns and duration can be utilized to obtain large scale data on sleep patterns and duration at the population,^96^ while artificial intelligence can help explore combined data sources and uncover hidden patterns.^96^, ^97^ In sophisticated settings, OMICS technology and single cell sequencing can reveal cellular pathways involved in sleep and immunity.^98^ Furthermore, new brain imaging techniques may reveal how sleep, inflammation and immunity interact within the brain.^99^, ^100^, ^101^, ^102^ Eventually, new insights should be translated into impact on the health of patients and healthy individuals, and new communication tools can be utilized to promote sleep hygiene.^103^ However, despite the potential of new technologies, each method faces specific challenges, for instance regarding data quality, quantity, and interpretability. Furthermore, high costs may limit the utilization of new techniques in clinical practice. The potentials and limitations of several new fields are summarized in Table 1.

**Table 1:** Potentials and limitations of new advances in sleep, inflammation, immunity and infections.

Technique/field	Potentials	Limitations	References
Wearable devices and sleep monitoring	Wearable sleep trackers, such as smartwatches and rings, can enable the collection of large‐scale data on sleep patterns and sleep quality, which can be linked to markers of immunity and infections.	Poor quality and inconsistency of devices and algorithms may lead to inaccurate results.	Goergen et al.^96^
Artificial intelligence (AI) and machine learning	AI enables the linkage of different datasets and the detection of hidden patterns and correlations. Thereby, AI may help predicting immune dysregulation and course of infections, which could lead to personalized approach in the clinical setting. AI model can simulate how sleep, immunity and inflammation interact during infections.	Data bias may lead to inaccurate results Interpretability can be complicated, which complicated the validation of findings and their application in clinical practice.	Watson et al.^97^
Omics technology, including single‐cell sequencing	Genomics, transcriptomics, proteomics and metabolomics may help uncover molecular pathways that are influenced by sleep and involved in immune regulation.	Large amount of data requireds sophisticated analytics to process and interpret data. The interpretation of functional significance of findings can be challenging. High costs currently limit the utilization of OMICS technologies, especially in the clinical setting.	Chen et al.^98^
Neuro‐immunology and brain imaging	Several new techniques, such as functional magnetic resonance imaging (fMRI), Magnetic resonance spectroscopy (MRS) and spatial transcriptomics, may help to uncover how sleep, inflammation and immunity interacts within the brain.	Advanced brain imaging technologies typically require subjects to be in a controlled environment, which may limit the relevance of findings to real‐world conditions. High costs limit the utilization of highly sophisticated neuroimaging technologies.	Raimondo et al.^101^ Moehlman et al.^100^ Vanrobaeys et al.^101^ Quintana et al.^102^
Lifestyle and sleep hygiene	New insights in lifestyle challenges and can guide the advocacy of sleep hygiene, while new communication tools can be utilized in outreaching to the target population.	The translation of new insights at the cellular or system level of inflammation and immunity to the clinical setting may be challenging.	Ramar et al.^103^

## 2 DISCUSSION

Sleep is primarily regarded as a state of the CNS, but the interactions with other physiological systems, including immunity, are increasingly recognized.^104^, ^105^ Although mechanisms are being revealed at the cellular and molecular level, as summarized in this review of literature, the evolutionary benefits of circadian regulation of immunity yet remain to be unsolved. Differential exposure to pathogens during sleep and when awake as well as homeostatic mechanisms may be involved.^16^, ^86^ It has been hypothesized that sleep regulates plasticity not only at the neural level of memory, but also in the cellular memory of specific immunity.^106^

Despite the importance of the link between sleep, immunity and infections in pathogenesis of disease, the direct implications on clinical practice are presently limited. For instance, infections are usually not differently treated in the presence or absence of a sleeping disorder. Understanding the link between sleep, inflammation, immunity and infections may help the clinician to identify disturbed sleep as an underlying factor in patients with inflammatory disease or repeated infections and promote interventions to improve sleep. Due to the link between inflammation and sleep disturbances, polysomnography for diagnosis of chronic sleep disorders should be avoided during acute disease, as the findings may not reflect sleep quality and architecture in the stable situation. Furthermore, the evidence reviewed here suggests that treating inflammation may improve sleep in a subset of patients.

Despite the increased understanding of immunity and inflammation in health and disease, measurement of cytokines and other markers have only limitedly been implemented in routine diagnostics in some diseases but not in sleep disturbances. Prospective studies linking markers of immunity before and during treatment of sleep disturbances could identify which markers can be useful in medical practice, and how.

The current knowledge of the role of immunity and inflammation in sleep disturbances and infections provide rationale for intervention with biologicals, which has been successful at least in some cases as demonstrated in this review. Therefore, it seems tempting to develop diagnostic and therapeutic tools directed at cytokines. However, social and behavioural factors could be more feasible for interventions, although clinical evidence on these interventions are limited, partly due to practical issues, such as funding model, the need for personalised approaches, and restricted possibility of blinding, which limit the application of lifestyle interventions in trials. Thus, an individualized approach should be developed to identify in which patients' lifestyle interventions are not effective and interventions should be personalized. This could prevent medicalisation and increase of costs associated with biological therapies.

## 3 CONCLUSION

Sleep is characterized by an enhanced Th1‐mediated inflammation in the early phase, and by transitioning to a regulating Th2 response dominated immunity in late sleep. Chronic sleep disturbances are associated with an elevated risk of infections and enhanced inflammation, which may have impact on other inflammatory comorbidities. Conversely, infections and inflammation can also disrupt sleep patterns and architecture. New technologies have the potential to accelerate our understanding of immune imbalance related to sleep disturbances. By recognizing sleep disturbances as both a contributor to and a consequence of immune dysregulation, clinicians can intervene to mitigate inflammatory conditions, which could ultimately contribute to better outcomes.

## CONFLICT OF INTEREST STATEMENT

In the past 3 years, the author received payments from Pfizer (lecture), BioMerieux (advisory board), Sanofi (advisory board), and from AstraZeneca (scientific consultant).

## Data Availability

Not applicable.
